# *Shigella* Detection and Molecular Serotyping With a Customized TaqMan Array Card in the Enterics for Global Health (EFGH): *Shigella* Surveillance Study

**DOI:** 10.1093/ofid/ofad574

**Published:** 2024-03-25

**Authors:** Jie Liu, Paul F Garcia Bardales, Kamrul Islam, Sheikh Jarju, Jane Juma, Chimwemwe Mhango, Queen Naumanga, Sonia Qureshi, Catherine Sonye, Naveed Ahmed, Fatima Aziz, Md Taufiqur Rahman Bhuiyan, Mary Charles, Nigel A Cunliffe, Mahamadou Abdou, Sean R Galagan, Ensa Gitteh, Ibrehima Guindo, M Jahangir Hossain, Abdoulie M J Jabang, Khuzwayo C Jere, Flywell Kawonga, Mariama Keita, Noumou Yakhouba Keita, Karen L Kotloff, Wagner V Shapiama Lopez, Stephen Munga, Maribel Paredes Olortegui, Richard Omore, Patricia B Pavlinac, Firdausi Qadri, Farah Naz Qamar, S M Azadul Alam Raz, Laura Riziki, Francesca Schiaffino, Suzanne Stroup, Sarata Nassoun Traore, Tackeshy Pinedo Vasquez, Mohammad Tahir Yousafzai, Martin Antonio, Jennifer E Cornick, Furqan Kabir, Farhana Khanam, Margaret N Kosek, John Benjamin Ochieng, James A Platts-Mills, Sharon M Tennant, Eric R Houpt

**Affiliations:** School of Public Health, Qingdao University, Qingdao, China; Asociación Benéfica PRISMA, Iquitos, Loreto, Peru; Infectious Diseases Division, International Centre for Diarrhoeal Disease Research, Bangladesh (icddr,b), Dhaka, Bangladesh; Medical Research Council Unit The Gambia, London School of Hygiene and Tropical Medicine, Fajara, The Gambia; Centre pour le Développement des Vaccins du Mali (CVD-Mali), Bamako, Mali; Malawi Liverpool Wellcome Research Programme, Blantyre, Malawi; Division of Infectious Diseases and International Health, University of Virginia, Charlottesville, Virginia, USA; Department of Pediatrics and Child Health, The Aga Khan University, Karachi, Pakistan; Center for Global Health Research, Kenya Medical Research Institute, Kisumu, Kenya; Department of Pediatrics and Child Health, The Aga Khan University, Karachi, Pakistan; Department of Pediatrics and Child Health, The Aga Khan University, Karachi, Pakistan; Infectious Diseases Division, International Centre for Diarrhoeal Disease Research, Bangladesh (icddr,b), Dhaka, Bangladesh; Malawi Liverpool Wellcome Research Programme, Blantyre, Malawi; Institute of Infection, Veterinary and Ecological Sciences, Department of Clinical Infection, Microbiology and Immunology, University of Liverpool, Liverpool, United Kingdom; Centre pour le Développement des Vaccins du Mali (CVD-Mali), Bamako, Mali; Department of Global Health, University of Washington, Seattle, Washington, USA; Medical Research Council Unit The Gambia, London School of Hygiene and Tropical Medicine, Fajara, The Gambia; Centre pour le Développement des Vaccins du Mali (CVD-Mali), Bamako, Mali; Medical Research Council Unit The Gambia, London School of Hygiene and Tropical Medicine, Fajara, The Gambia; Medical Research Council Unit The Gambia, London School of Hygiene and Tropical Medicine, Fajara, The Gambia; Malawi Liverpool Wellcome Research Programme, Blantyre, Malawi; Institute of Infection, Veterinary and Ecological Sciences, Department of Clinical Infection, Microbiology and Immunology, University of Liverpool, Liverpool, United Kingdom; Department of Medical Laboratory Sciences, School of Life Sciences and Health Professions, Kamuzu University of Health Sciences, Blantyre, Malawi; Malawi Liverpool Wellcome Research Programme, Blantyre, Malawi; Medical Research Council Unit The Gambia, London School of Hygiene and Tropical Medicine, Fajara, The Gambia; Centre pour le Développement des Vaccins du Mali (CVD-Mali), Bamako, Mali; Center for Vaccine Development and Global Health, University of Maryland School of Medicine, Baltimore, Maryland, USA; Department of Medicine, University of Maryland School of Medicine, Baltimore, Maryland, USA; Department of Pediatrics, University of Maryland School of Medicine, Baltimore, Maryland, USA; Asociación Benéfica PRISMA, Iquitos, Loreto, Peru; Center for Global Health Research, Kenya Medical Research Institute, Kisumu, Kenya; Asociación Benéfica PRISMA, Iquitos, Loreto, Peru; Center for Global Health Research, Kenya Medical Research Institute, Kisumu, Kenya; Department of Global Health, University of Washington, Seattle, Washington, USA; Infectious Diseases Division, International Centre for Diarrhoeal Disease Research, Bangladesh (icddr,b), Dhaka, Bangladesh; Department of Pediatrics and Child Health, The Aga Khan University, Karachi, Pakistan; Infectious Diseases Division, International Centre for Diarrhoeal Disease Research, Bangladesh (icddr,b), Dhaka, Bangladesh; Center for Global Health Research, Kenya Medical Research Institute, Kisumu, Kenya; Division of Infectious Diseases and International Health, University of Virginia, Charlottesville, Virginia, USA; Faculty of Veterinary Medicine, Universidad Peruana Cayetano Heredia, Lima, Peru; Division of Infectious Diseases and International Health, University of Virginia, Charlottesville, Virginia, USA; Centre pour le Développement des Vaccins du Mali (CVD-Mali), Bamako, Mali; Asociación Benéfica PRISMA, Iquitos, Loreto, Peru; Department of Pediatrics and Child Health, The Aga Khan University, Karachi, Pakistan; Medical Research Council Unit The Gambia, London School of Hygiene and Tropical Medicine, Fajara, The Gambia; Centre for Epidemic Preparedness and Response, London School of Hygiene & Tropical Medicine, London, UK; Department of Infection Biology, Faculty of Infectious and Tropical Diseases, London School of Hygiene & Tropical Medicine, London, UK; Malawi Liverpool Wellcome Research Programme, Blantyre, Malawi; Institute of Infection, Veterinary and Ecological Sciences, Department of Clinical Infection, Microbiology and Immunology, University of Liverpool, Liverpool, United Kingdom; Department of Pediatrics and Child Health, The Aga Khan University, Karachi, Pakistan; Infectious Diseases Division, International Centre for Diarrhoeal Disease Research, Bangladesh (icddr,b), Dhaka, Bangladesh; Division of Infectious Diseases and International Health, University of Virginia, Charlottesville, Virginia, USA; Center for Global Health Research, Kenya Medical Research Institute, Kisumu, Kenya; Division of Infectious Diseases and International Health, University of Virginia, Charlottesville, Virginia, USA; Center for Vaccine Development and Global Health, University of Maryland School of Medicine, Baltimore, Maryland, USA; Department of Medicine, University of Maryland School of Medicine, Baltimore, Maryland, USA; Division of Infectious Diseases and International Health, University of Virginia, Charlottesville, Virginia, USA

**Keywords:** *ipaH*, PCR, quantification, rectal swab, serotyping

## Abstract

**Background:**

Quantitative polymerase chain reaction (qPCR) targeting *ipaH* has been proven to be highly efficient in detecting *Shigella* in clinical samples compared to culture-based methods, which underestimate *Shigella* burden by 2- to 3-fold. qPCR assays have also been developed for *Shigella* speciation and serotyping, which is critical for both vaccine development and evaluation.

**Methods:**

The Enterics for Global Health (EFGH) *Shigella* surveillance study will utilize a customized real-time PCR–based TaqMan Array Card (TAC) interrogating 82 targets, for the detection and differentiation of *Shigella* spp, *Shigella sonnei*, *Shigella flexneri* serotypes, other diarrhea-associated enteropathogens, and antimicrobial resistance (AMR) genes. Total nucleic acid will be extracted from rectal swabs or stool samples, and assayed on TAC. Quantitative analysis will be performed to determine the likely attribution of *Shigella* and other particular etiologies of diarrhea using the quantification cycle cutoffs derived from previous studies. The qPCR results will be compared to conventional culture, serotyping, and phenotypic susceptibility approaches in EFGH.

**Conclusions:**

TAC enables simultaneous detection of diarrheal etiologies, the principal pathogen subtypes, and AMR genes. The high sensitivity of the assay enables more accurate estimation of *Shigella*-attributed disease burden, which is critical to informing policy and in the design of future clinical trials.

## Teaser

*Shigella* spp, *Shigella flexneri* serotypes, and other diarrhea-associated enteropathogens are detected and quantified with a customized real-time polymerase chain reaction–based TaqMan Array Card in the Enterics for Global Health: *Shigella* surveillance study. *Shigella* burden will be estimated with a quantitative methodology.

The genus *Shigella* consists of 4 species: *S. dysenteriae*, *S. flexneri*, *S. boydii*, and *S. sonnei* [1]. Globally, *S. flexneri* is the most predominant species, accounting for approximately 60% of *Shigella* infections in low- and middle-income countries, and *S. sonnei* is the second most common, responsible for an estimated 10%–20% of *Shigella* infections in such settings [2–4]. *Shigella flexneri* possesses at least 19 known different serotypes based on the structure of its surface lipopolysaccharide O-antigen [5]. Traditionally, culture-based methods and biochemical properties have been used to isolate and differentiate *Shigella* spp from clinical samples [6]. The Enterics for Global Health (EFGH): *Shigella* surveillance study will perform molecular testing for *Shigella* as an adjunct to culture for several reasons [7]. First, stool culture has limited sensitivity for detecting bacterial enteropathogens, including *Shigella*, and takes 2–3 days [8]. Culture is also challenging as *Shigella* requires stringent sampling, transport, and growth conditions for optimal recovery and can also be sensitive to changes in pH [9], temperature, and oxygen levels [10]. Delayed placement in transport media, prolonged transport times, and unstable cold chain may compromise the yield. Additionally, *Shigella* can be easily overgrown by other bacteria, leading to difficulties in its isolation and identification [11]. Last, culture methods may yield false-negative results due to the low bacterial load of *Shigella* present in clinical specimens [12], or antibiotic use prior to seeking care/sample collection [13]—a more common practice in some of the EFGH sites [14, 15].

The current *Shigella* vaccine candidates mostly target particular *S. flexneri* serotypes and *S. sonnei*; therefore, speciation and serotyping are critical for both vaccine development and evaluation. Conventionally, serotyping of *Shigella* isolates by antisera agglutination has been used as the standard method. This method is time consuming, expensive, and sometimes inaccurate due to variation in performance of antisera produced by different companies or unavailability of antisera [16]. Test interpretation requires visual assessment of agglutination reactions and an interpretation scheme that can be ambiguous. In addition, conventional antisera serotyping must be done on pure isolates and cannot be done directly on stool. These deficiencies led to the development of polymerase chain reaction (PCR) serotyping assays for *S. flexneri* targeting specific *gtr* and *oac* O-antigen modification genes [5, 17–20], which will be utilized in this study.

After *Shigella* or other pathogens are detected by molecular methods, another important component to consider is disease attribution. For instance, coinfections are common in resource-limited settings [21, 22]. Previous studies using molecular detection methods detected >3 pathogens in >70% of children presenting with diarrhea in Malawi [23], up to 5 pathogens coexisting in 1 single specimen in a Bangladeshi population [24], and up to 6 pathogens in India [25]. A few studies have suggested that coinfections may cause worse clinical outcomes [26–28].

Therefore, to detect *Shigella*, provide speciation and serotyping data, and test for multiple enteropathogens, we have chosen to use the 384-well microfluidic TaqMan Array Card (TAC) as the diagnostic platform. The platform has high sensitivity and specificity compared to other singleplex real-time PCR systems and offers simplified operational procedures [29–31]. Numerous studies have validated the use of this technique and shown it to be robust across laboratories and sample types [31–37]. TAC provides flexibility to adjust for targets, a strategy that would not be possible if commercially designed platforms for enteric multiplex assays were employed. Leveraging the rich pathogen data, in secondary analysis we also intend to explore the impact of coinfection on burden and consequences.

## METHODS

### Protocol Development and Training

Standard operating procedures were adapted from previous studies and reviewed by representatives from all EFGH participating sites during monthly EFGH laboratory working group meetings that occurred during the 12-month planning phase of EFGH. A 5-day on-site training was conducted by a research scientist from the University of Virginia (UVA) at the beginning of the study at each of the 7 EFGH recruiting sites. It included review and hands-on practice of all study protocols, from sample extraction to data analysis and data management. The training also covered instrument calibration and maintenance and preparing the laboratory environment for molecular testing. Proficiency testing of all laboratory team members who would be performing the assay for the study was done either during the training for bench activities by assaying the external quality assessment (EQA) samples or after the training for data analysis by analyzing the EQA data files. Follow-up site visits are scheduled on an annual basis or per site request, with Zoom refresher trainings or troubleshooting emails occuring any time the need arises.

### Sample Collection

Rectal swabs (Pediatric FLOQswab®, Copan Diagnostics) have been chosen as the primary specimen collection modality in the EFGH study. In healthcare-based settings, rectal swabs allow for a high specimen collection rate across study sites and will facilitate a shorter time period between collection and storage or placement into transport media for stool culture [38]. Rectal swab samples, unsurprisingly given their smaller stool volume, generally showed a higher quantification cycle (Cq) in quantitative PCR (qPCR) for most targets versus the corresponding whole stool [37, 39–41]. A good correlation was observed for Cq values between paired swab and stool samples, with swab Cqs usually 1–3 cycles higher than stool. Nonetheless, as a Cq of 35 is used as the analytical cutoff of TAC, and diarrhea-associated Cq cutoffs on stool are generally <30 [21, 42], swabs are sufficiently sensitive to detect diarrhea-associated pathogens. To confirm the correlation between rectal swabs and stool, and quantitative detection differences between the 2 specimen types, 2 of the 7 EFGH study sites (Bangladesh and The Gambia) will collect paired swab and stool samples for an internal swab-stool comparison substudy to refine the pathogen-specific Cq conversion between swab and stool. All caregivers of children participating in this study will provide parental consent following the informed consent process and provide written informed consent prior to any study procedures.

### Total Nucleic Acid Extraction

Upon sample collection, the bottom flocked portion of the rectal swab is stored frozen at −80°C in a 2-mL Sarstedt (Sarstedt, Nümbrecht, Germany) tube after the shaft of the swab is snapped off. For whole stool, 200 mg (180–220 mg) or 200 µL if watery is aliquoted into the same type of Sarstedt tube that is compatible with a bead beater. Total nucleic acid is extracted directly from stored rectal swab or stool samples using a modified QIAamp Fast DNA Stool mini kit (Qiagen, Hilden, Germany) [37] with pretreatment, including bead beating and 95°C incubation to increase the yield. Nucleic acid is then eluted with 200 μL of elution buffer (ATE). External controls, 10^6^ phocine herpes virus (PhHV) and 10^7^ MS2 bacteriophage, are spiked into each sample during the initial lysis step to monitor the extraction and amplification efficiency. One extraction blank is included per batch of extraction to monitor contamination.

### TAC Setup

TAC is a real-time PCR system consisting of 384 wells that allows the simultaneous processing of 8 samples, each of which can be tested for 48 targets or more if duplex tests with different fluorophores are employed [43]. The qPCR primers and probes were derived from previous research [36, 37] and are manufactured along with the card. In this study, 82 targets were selected (Table 1), including genomic targets from bacteria, viruses, and parasites, *Shigella* speciation (for *S. flexneri* and *S. sonnei* only) and serotyping targets (for *S. flexneri* only), colonization factors of enterotoxigenic *Escherichia coli*, and gene targets associated with antimicrobial resistance (AMR), in addition to 2 external controls (MS2 and PhHV). qPCR reactions are performed with the Ag-Path-ID One Step RT-PCR kit (Life Technologies, Carlsbad, California). The master mix is prepared by mixing 425 µL of Ag-Path-ID 2X RT-PCR buffer and 34 µL Ag-Path-ID enzyme mix, then 54 µL aliquoted into 8 tubes. Forty-six microliters of the total nucleic acid extract from swab (or extraction blank, or nuclease-free water as no template control) or 20 µL from stool (supplemented with 26 µL of nuclease-free water) are added to each tube. The mixture is loaded into the TAC card following the manufacturer's instruction. The TAC card is then loaded onto the ViiA 7 or QuantStudio 7 real-time PCR instrument (Life Technologies) and analyzed using QuantStudio real-time PCR software. The qPCR experiment is set up with a template pre-populated with layout, cycling conditions, assay thresholds, and flag setting, etc. qPCR is programmed to run under the following conditions: 45°C for 20 minutes and 95°C for 10 minutes, followed by 40 cycles of 95°C for 15 seconds and 60°C for 1 minute.

**Table 1.** Quantitative Polymerase Chain Reaction Targets to Be Used in the Enterics for Global Health: *Shigella* Surveillance Project

Target Type	Target	Gene
Virus	Adenovirus 40/41	Fiber
	Astrovirus	Capsid
	Norovirus GI	ORF1/ORF2
	Norovirus GII	ORF1/ORF2
	Rotavirus	*NSP3*
	Sapovirus	*RdRp*
Bacteria	*Aeromonas*	Aerolysin
	*Campylobacter jejuni/coli*	*cadF*
	*Helicobacter pylori*	*ureC*
	*Plesiomonas*	*gyrB*
	*Salmonella enterica*	*ttr*, *invA*
	*Shigella/*Enteroinvasive *E. coli*	*ipaH*
	*Vibrio cholerae*	*hlyA*
	Enteroaggregative *E. coli*	*aaiC*, *aatA*
	Enteropathogenic *E. coli*	*bfpA*, *eae*
	Enterotoxigenic *E. coli*	*LT*, *STh*, *STp*
	Shiga toxin–producing *E. coli*	*Stx1*, *Stx2*
	ETEC colonization factor	CFA/I, CS1, CS2, CS3, CS5, CS6
	*Shigella flexneri* serotyping^a^	*gtrI*, *gtrIc*, *gtrII*, *gtrIV*, *gtrV*, *gtrX*, *oac*, *wzx6*
	*Shigella sonnei*	*Rhs*, *pm*
	Macrolide resistance	*ermA*, *ermB*, *ermC*, *mphA*, *mphB*, *mefA*, *msrA*, *msrD*
	Fluoroquinolone resistance	*Shigella/E. coli* gyrA S83L
	Polymyxin resistance	*MCR1*, *MCR2*
	β-lactam resistance	CTX-M M1, M2, M74, M8, M25, M9
		OXA48
		SHV, SHV23840
		TEM E104K, R164SC, G238S
Fungus	*Enterocytozoon bieneusi*	ITS
Protozoa	*Cryptosporidium*	18S rRNA
	*Entamoeba histolytica*	18S rRNA
	*Giardia lamblia*	18S rRNA
	*Cyclospora cayetanensis*	18S rRNA
	*Cystoisospora belli*	18S rRNA
External control	MS2	*MS2g1*
	PhHV	*gB*

The primers and probes designed are adapted from previous studies [17, 18, 34, 44, 45].

Abbreviations: ETEC, enterotoxigenic *Escherichia coli*; MS2, MS2 bacteriophage; PhHV, phocine herpes virus; rRNA, ribosomal RNA.

^a^ Table 2 shows the serotyping scheme with these gene targets.

### Run Analysis

With QuantStudio real-time PCR software, amplification curves are examined target by target, and baselines are adjusted as needed to correct false-positive/negative or inaccurate Cq values. It is required that each file be examined sequentially by 2 individuals. The results are exported into an Excel file when all of the targets are examined and adjusted to satisfaction. The export file is uploaded onto the MuSIC (Multi-Schema Information Capture) database housed at UVA [46]. An automated TAC analysis program is also under development and may be used to speed these manual run analyses.

### Data Quality Control

Four types of controls are incorporated throughout the testing procedure: TAC positive control, no template control, external controls, and extraction blank. The TAC positive control combines synthetic constructs containing the concatenated target fragments (plasmid for DNA targets and in vitro transcripts for RNA viruses) [36]. A 10-fold serial dilution of TAC positive control is prepared and run at 3 replicates every 6 months (or after instrument maintenance or repair). This serves as a performance check and generates standard curves to derive copy numbers from Cqs if needed. A no template control (ie, nuclease-free water) is run every 10 cards to monitor for qPCR reagent contamination.

A Cq of 35 is set as the analytical cutoff for the pathogen targets. External controls and extraction blanks are used to validate the negative and positive results, respectively. Specifically, the negative results (no amplification or Cq >35) of a sample are valid only when the external controls amplify with Cq <35 (PhHV for DNA targets, MS2 for RNA targets). The positive results of a sample are valid only when the extraction blank that is extracted along with the sample is negative for the relevant targets. Otherwise, the results are deemed to be invalid, and excluded from data analysis.

Fluorescence fluctuation during qPCR is monitored by the system and reflected in the quality control (QC) summary of QuantStudio real-time PCR software. The QC items BADROX (bad passive reference signal) combined with NOISE (noise higher than others in plate) or SPIKE (noise spikes) have been found to affect the accuracy of the results; thus, any data with these flags are determined to be invalid.

The laboratory surfaces and equipment used for sample processing are periodically tested using a swipe test kit provided centrally to determine the potential source of contamination. Swipe testing and cleaning/decontamination procedures should occur after any pathogen target is detected in an extraction blank or no template amplification control.

### Quality Assessment

Stool samples for the EQA are prepared at UVA by spiking a combination of bacterial, viral, and protozoan targets at various concentrations into stool samples from healthy donors, then shipping blinded samples to the study sites on dry ice. Bacterial culture and commercial *Cryptosporidium* oocysts are spiked directly into stool, followed by incubation at 95°C for 30 minutes to inactivate the infectious agents. In vitro transcripts for RNA viruses are lyophilized and spiked into the Inhibitex buffer during extraction. One set of 5 EQA samples is tested at each study site every 6 months. The test results are evaluated by the UVA laboratory and 80% concordance is required prior to testing clinical samples. Additionally, UVA provides TAC run files for data analysis EQA to evaluate the accuracy of the test results. All of the laboratory personnel performing TAC testing are trained by a UVA scientist on the entire procedure and are required to pass their proficiency tests before performing their own sample runs.

### Data Analysis

For EFGH, Cq cutoffs will be used to determine the likely attribution of particular etiologies of diarrhea, leveraging previous studies that performed qPCR testing of both diarrheal and nondiarrheal stools, specifically the 7-site Global Enteric Multicenter Study (GEMS) and the 8-site Malnutrition and the Consequences for Child Health and Development (MAL-ED) cohort study [21, 42]. Using models identical to those used in those studies but limited to children <36 months of age, quantity-specific odds ratios were estimated from each of GEMS and MAL-ED independently for Cq values ranging from 35 to 15 by 0.001 increments by taking the median odds ratio from 10 000 random permutations of the model coefficients, drawn equally from each of the site-specific models. For each quantity, the episode-specific attributable fraction (AFe) was then calculated, where $AFe_{i}=1/j*(1\text{–}1/OR_{i})$, and *OR_i_* is the quantity-specific median odds ratio. A LOESS regression was fitted and the highest Cq value with an AFe ≥0.5 (ie, majority attribution) was picked. Finally, in the case that a cutoff was derived from both studies, the mean Cq value was calculated, and if a cutoff was identified in only 1 of GEMS and MAL-ED, that cutoff from that single study was used directly (Table 3). To account for the lower sensitivity of rectal swab, we applied a correction to determine swab-specific cutoffs, also outlined in Table 3.

**Table 2.** *Shigella flexneri* Serotyping Scheme for the Enterics for Global Health: *Shigella* Surveillance Project

*Shigella* Serotype	Gene Target
*S. flexneri* 1a	*gtrI*
*S. flexneri* 1b	*gtrI*, *oac*
*S. flexneri* 1d	*gtrI*, *gtrX*
*S. flexneri* 2a	*gtrII*
*S. flexneri* 2b	*gtrII*, *gtrX*
*S. flexneri* 3a	*gtrX*, *oac*
*S. flexneri* 3b	*oac*
*S. flexneri* 4a	*gtrIV*
*S. flexneri* 4b	*gtrIV*, *oac*
*S. flexneri* 5a	*gtrV*, *oac*
*S. flexneri* 5b	*gtrV*, *gtrX*, *oac*
*S. flexneri* 6	*wzx6*
*S. flexneri* 7a	*gtrI*, *gtrIc*
*S. flexneri* X	*gtrX*

**Table 3.** Diarrhea-Associated Quantification Cycle Cutoffs Derived From the Global Enteric Multicenter Study and Malnutrition and the Consequences for Child Health and Development Study

Pathogen	Quantification Cycle Cutoff			
	Whole Stool			Rectal Swab
	GEMS	MAL-ED	EFGH^a^	EFGH^b^
Adenovirus 40/41	24.7	22.3	23.5	25.5
*Aeromonas*	21.2	22	21.6	23.6
Astrovirus	23.8	24.9	24.4	26.7
*Campylobacter jejuni/coli*	None	19.9	19.9	20.7
*Cryptosporidium*	26.9	23.7	25.3	27.1
*Cyclospora cayetanensis*	31.9	None	31.9	33.9
*Entamoeba histolytica*	31.3	29.8	30.6	32.6
*Cystoisospora belli*	None	32.4	32.4	34.4
Norovirus GII	20.6	27.7	24.2	26.6
Rotavirus	32.3	31.3	31.8	34.2
*Salmonella*	31.3	None	31.3	33.3
Sapovirus	17.1	26.8	21.9	24.4
*Shigella*/EIEC	29.4	30.1	29.8	31.1
ST-ETEC	23	25.2	24.2	27.9
Typical EPEC	None	17.4	17.4	20.7
*Vibrio cholerae*	33.4	30.3	31.9	33.9

Abbreviations: EFGH, Enterics for Global Health; EIEC, enteroinvasive *Escherichia coli*; EPEC, enteropathogenic *Escherichia coli*; GEMS, Global Enteric Multicenter Study; MAL-ED, Malnutrition and the Consequences for Child Health and Development; ST-ETEC, enterotoxigenic *Escherichia coli* producing heat-stable enterotoxins.

^a^Calculated as the mean of GEMS and MAL-ED whole stool quantification cycle value cutoffs.

^b^Calculated as the mean of GEMS and MAL-ED Cq cutoffs plus the rectal swab conversion. Note that the Bangladesh and The Gambia sites will be providing additional swab-stool comparative data that could slightly alter the swab adjustment.

For *Shigella*, previous studies have determined a diarrhea-associated *Shigella* amount of approximately 10^7^ or more copies of the *ipaH* gene per gram of stool, equivalent to an *ipaH* Cq of 29 with TAC [21], and the cutoff derived for EFGH (stool 29.8, swab 31.1) is extremely close to this. For pathogen assays, a Cq of 35 will be used as the limit of detection, as we have previously shown that detections on the TAC platform with a Cq >35 are at the limit of detection and not reproducible [31]. Primary analyses will ignore other attributable etiologies; therefore, a child with *Shigella* at or below the etiologic cutoff will be considered to have attributable *Shigella*. In secondary analyses, *Shigella* molecular data will be stratified by presence/absence of 1 or more other pathogens at or below a Cq threshold. Also in secondary analyses, attributable pathogens will be reported using a standard Cq attribution cutoff of 30 across the pathogens, a cutoff that adds specificity to the sensitive molecular assay without conditioning on previous data to arrive at pathogen-specific cutoffs.

To identify *Shigella* species and serotypes, we will consider all samples with a swab *ipaH* Cq <31.1. Then additionally we will require the following [20]:

1. The Cq of the *S. flexneri* serotyping target or *S. sonnei* target must be within 7 Cq of the *ipaH* Cq (ie, Cq values of up to 38.1).
2. If ≥2 targets are required to determine the serotype, the Cq difference between the targets must be ≤2 Cq.
3. If multiple *S. flexneri* serotypes and/or *S. sonnei* are detected using the above criteria, the target(s) with the lower Cq determines the primary species present.
4. This algorithm will be compared to culture and may be refined. For example, the Bangladesh and The Gambia sites will be providing additional swab-stool comparative data that could slightly alter the swab adjustment.

## DISCUSSION

Here we have described the rationale and methodology that will be used for molecular detection of *Shigella* and attribution of etiology. Of note, the PCR target for *Shigella* has typically been *ipaH* [47]. *Shigella* possesses 12 unique invasion plasmid antigen H (*ipaH*) genes [48], which are important for pathogenesis by encoding proteins used to evade the host immune response during infection [49–51]. These genes are present in all 4 *Shigella* spp as well as enteroinvasive *E. coli* (EIEC) [44]. Therefore, while the *ipaH* gene cannot differentiate *Shigella* from EIEC, the prevalence of EIEC has typically been much lower [45], and metagenomic sequencing results indicated that *ipaH* qPCR-positive samples are similar to those of *Shigella* culture-positive samples in *Shigella* sequence composition, supporting *ipaH* qPCR as an accurate method for detecting *Shigella* [52]. Numerous studies have demonstrated *ipaH* qPCR to be highly efficient in detecting *Shigella* in clinical samples compared to culture-based methods, which underestimated *Shigella* burden by 2- to 3-fold [21, 43, 53, 54].

The 82 targets interrogated in this study cover the main diarrhea-associated enteropathogens, important pathogen subtyping targets, and AMR genes. As for AMR genes, we will evaluate genotypic resistance markers directly in rectal swab/stool for 4 classes of antibiotics, including fluoroquinolones, macrolides, polymyxin, and for β-lactamases. The gene targets were chosen based on previously reported genes or mutations [13, 55]. Because AMR genes are often shared between bacteria on plasmids, studies have shown that the molecular detection of drug resistance genes in stools does not implicate a particular organism. That said, a study of *Shigella* treatment showed that the lack of detection of macrolide resistance genes *mphA* or *ermB* genes has a high negative predictive value for macrolide resistance [13]. In this study we will be able to further compare the conventional susceptibility results performed on the *Shigella* isolates with that found in stool.

In summary, the TAC approach allows for the improved detection of *Shigella* relative to the historic standard of culture and comparable to that of other nucleic acid detection systems. Furthermore, the method allows for speciation and serotyping of *Shigella*, detection of other pathogens, and detection of AMR genes in one run. This combination of diagnostic characteristics will be directly compared to traditional culture, serotyping, and phenotypic susceptibility approaches in EFGH and will provide critical data for subsequent field studies.

## Notes

***Author contributions*.** J. L., P. F. G. B., K. I., S. J., J. J., C. M., Q. N., S. Q., C. S., M. A., J. E. C., F. Kab., F. Kh., M. N. K., J. B. O., J. A. P.-M., S. M. T., and E. R. H. actively participated in monthly working group meetings during which the conceptualization and outline was discussed and agreed upon. J. L., P. F. G. B., K. I., S. J., J. J., C. M., Q. N., S. Q., and C. S. wrote the first draft of the manuscript, and M. A., J. E. C., F. Kab., F. Kh., M. N. K., J. B. O., J. A. P.-M., S. M. T., and E. R. H. provided review, scientific input, and editing. N. A., F. A., M. T. R. B., M. C., N. A. C., M. A., S. R. G., E. G., I. G., M. J. H., A. M. J. J., K. C. J., F. Kaw., M. K., N. Y. K., K. L. K., W. V. S. L., S. M., M. P. O., R. O., P. B. P., F. Q., F. N. Q., S. M. A. A. R., L. R., F. S., S. S., S. N. T., T. P. V., and M. T. Y. reviewed and edited the manuscript. All authors approved the content of the final manuscript.

***Financial support***. This project is supported by the Bill & Melinda Gates Foundation (award numbers INV-028721, INV-041730, INV-016650, INV-031791, INV-036891, INV-036892) and the National Institutes of Health (award numbers D43TW010913 to M. N. K. and M. P. O. and K43TW012298 to F. S.). Medical Research Council Unit The Gambia at the London School of Hygiene and Tropical Medicine is supported by the United Kingdom Research and Innovation Medical Research Council (program number MC_UU_00031/1 and MC_UU_00031/5). Nigel A. Cunliffe is a National Institute for Health and Care Research (NIHR) Senior Investigator (NIHR203756). Nigel A. Cunliffe and Khuzwayo C. Jere are affiliated to the NIHR Global Health Research Group on Gastrointestinal Infections at the University of Liverpool; and to the NIHR Health Protection Research Unit in Gastrointestinal Infections at the University of Liverpool, a partnership with the UK Health Security Agency in collaboration with the University of Warwick. The views expressed are those of the author(s) and not necessarily those of the NIHR, the Department of Health and Social Care, the UK government or the UK Health Security Agency.

***Supplement sponsorship.*** This article appears as part of the supplement “Enterics for Global Health (EFGH) *Shigella* Surveillance Study-Rationale and Methods,” sponsored by the Bill & Melinda Gates Foundation.
